# 

**Published:** 1883-08

**Authors:** 


Answers to Practical Questions on the Subject of Diphtheria, from a Large Number of Replies to Questions by the *Therapeutic Gazette.*

The Bead Sature, a Modification of the Quill Sature for Palatoplasty, etc. By David Prince, M.D., of Jacksonville, Ill., also

A Practical Obturator, by the same author.

Hints on the Treatment of Some Parasitic Skin Diseases. By G. H. Rohe, M.D., Prof. of Hygiene and Clinical Dermatology, College of Physicians and Surgeons, Baltimore, Md.,—also

The Treatment of the Various Forms of Acne. By the same author.

On Nasal Cough and the Existence of a Sensative Reflex Area in the Nose. By John N. McKenzie, M.D., of Baltimore, Md., Surgeon of the Baltimore Eye, Ear and Throat Charity Hospital.

Antisepsis in Ovariotomy and Battey's Operation, Eighteen Consecutive Cases. All Successful. By Robert Beattey, M.D., of Rome, Ga. Also, by the same author,

A Monograph on Intra Uterine Medication by Iodized Phenol.

A Method Proposed to Secure Children Against Attacks of Diphtheria. By F. Peyre Porcher, M.D., of Charleston, S. C., Prof. of Materia Medica and Therapeutics in the Medical College of the State of South Carolina.

The doctor thus introduces his prescription.

In the course of a review published in the *Charleston Medical Journal* many years since, and also in a letter to a medical gentlemen in Philadelphia, written in 1879, which was copied in the *Medical Brief*, of St. Louis, Mo., I proposed a plan for preventing the attacks of this fatal disease. This was by measures to be used by those who were well, but who were exposed to its contagion. My ideas were based upon the fact that this disease (whether or not it depended upon the presence of a specific germ) at its inceptive stage was local, and generally had its seat in the fauces, which if acted on and modified by suitable agents, would not offer a nidus for its reception. If micro-organisms are the agents by which diphtheria is caused or propagated, then they will be less likely to effect a lodgement upon surfaces which are subjected to the repeated action of remedies which, whilst uninjurious, may also prove efficient in the destruction of such organisms. I also selected agents well known for their activity and value as tonics, depurants and antiseptics, and which would be adapted to the treatment of the diseases should our efforts at prevention prove abortive.

The prescription is as follows—the alcohol being a comparatively recent addition :

| | | |
|---|---|---|
| R | Tinct. of the chloride............ | 2 to 3 drams. |
| | Chlorate of potash............... | 2 to 3 drams. |
| | Quinine.... ..................... | 15 to 20 gr. |
| | Hyposulphite of soda............ | 1 to 2 drams. |
| | Alcohol........................... | 1 ounce. |
| | Water............................ | 6 ounces. |

M. S.—A teaspoon to a dessertspoonful two or three times a day in a little water—to be used by those exposed to the disease. The size of the dose may vary with the age of the subject and the nature of the exposure.

A NEW DISCOVERY, The Functions of the Spleen—Containing an Explanation of the Functions of the Spleen. Its Relations to the Circulation of the Blood and the Stomach, with Advanced Philosophical Views upon the Creation of Force and Motion in the Circulation of the Blood. By George H. Russell, of Newburg, Pa.

THE PHARMACEUTICAL RECORD.—This journal, formerly under the title of Martin's Chemists' and Druggists' Bulletin, now appears under above new name. It will be found a practical and reliable adjunct to the wants of the pharmacist, and worthy of his confidence and support. Address P. W. Bedford, P. O. Box 1807, New York City.

